# Global Patterns of Trends in Cholera Mortality

**DOI:** 10.3390/tropicalmed8030169

**Published:** 2023-03-13

**Authors:** Irena Ilic, Milena Ilic

**Affiliations:** 1 Faculty of Medicine, University of Belgrade, 11000 Belgrade, Serbia; 2 Department of Epidemiology, Faculty of Medical Sciences, University of Kragujevac, 34000 Kragujevac, Serbia

**Keywords:** cholera, epidemiology, mortality

## Abstract

Background: Cholera is a large public health issue, especially in countries with limited resources. The aim of the study was to determine trends in global cholera mortality from 1990–2019. Methods: This research is an observational, descriptive epidemiological study. The age-standardized rates (ASRs, per 100,000 population) of cholera mortality from 1990 to 2019 were evaluated through joinpoint regression analysis (by calculating Odds Ratio—OR, with corresponding 95% Confidence Interval—95% CI). Results: From 1990–2019 in the world, the number of deaths due to cholera in both sexes together increased, ranging from 83,045 in 1990 to 117,167 in 2019. During the observed period, there were about 3.0 million deaths due to cholera in the world. In both sexes together in 2019, the cholera mortality rate was the highest in Nigeria (ARS = 39.19) and Central African Republic (ARS = 38.80), followed by populations in Eritrea (ARS = 17.62) and Botswana (ARS = 13.77). Globally, cholera-related mortality significantly decreased in males (AAPC = −0.4%, 95% CI = −0.7 to −0.1), while a stable trend was noted in females (AAPC = −0.1%, 95% CI = −0.4 to 0.2) in the observed period. In the African Region, significantly increasing cholera-related mortality trends were observed both in males and females (AAPC = 1.3% and AAPC = 1.1%, respectively). Conclusions: Cholera mortality showed a constantly increasing trend in the African Region over the last three decades. More efforts in cholera management are necessary for effective response to the growing mortality in developing countries.

## 1. Introduction

Although ways of transmission, as well as the measures to prevent infection and control outbreaks, became well-known during the last 150 years, cholera still remains a significant public health problem in many countries in Africa, Asia, and South and Central America [1,2,3,4]. In addition to that, the current 7th pandemic of cholera (which started in South Asia in 1961, got to Africa in 1971, and the Americas in 1991) is still ongoing [1,5]. Today, cholera is endemic in many countries [1,2,6]. It was previously reported that cholera caused an estimated 95,000 deaths each year over the five years under study (2008–2012) [6]. In the second decade of the 20th century, cholera remained a significant problem globally; it occurs in the form of large outbreaks (such as those in Haiti and Yemen, Nigeria, the Democratic Republic of the Congo, the Dominican Republic, Egypt, Somalia, Bangladesh, Pakistan, Philippines, China, Ghana, Cameroon), and endemic diseases in many countries [1,2,4,5,6].

Cholera is a marker of inequality and affects the world’s poorest people, striking those who have already been made vulnerable by poverty and conflict [1,2,4,5,6]. Typical at-risk areas include those where basic infrastructure is not available and where access to clean water and adequate sanitation is lacking, as well as increasing humanitarian crises (camps for internally displaced persons or refugees, etc.), climate changes, multiple other disease outbreaks, sub-optimal/delayed surveillance (hindered response, insufficient laboratory capacity, use of heterogenous case definitions, etc.), delayed or depleted supply chain of medical commodities, limited availability of healthcare resources, availability of oral cholera vaccine [7,8].

Up to today, cholera remains a global threat to public health and is an indicator of inequity and lack of social development [4,6]. However, cholera remains a neglected and underreported disease [2,6]. The real burden of cholera is underreported [9]. The discrepancy between reported and estimated figures of the disease can be due to the fact that many cases are not recorded due to limitations in surveillance systems. Moreover, since 2005 notification of cases of cholera to the World Health Organization (WHO) is no longer mandatory [1]. The purpose of this study was to contribute data on trends in cholera-related mortality in the world.

## 2. Material and Methods

This study evaluated cholera-related mortality in the world from 1990 to 2019.

### 2.1. Study Design

A descriptive epidemiological study was carried out. Cholera-related mortality in the last three decades in the world was analyzed according to location, age, and sex. 

### 2.2. Data Source

Cholera-related mortality data were extracted from the Global Burden of Disease (GBD) 2019 study [10]. The GBD database provides a comprehensive assessment of cholera mortality across the world. The GBD 2019 study provides up-to-date data for mortality due to cholera for both sexes and for 204 countries and territories. The data were extracted from the number of cholera deaths reported to the WHO at the country-year level, vital registration data, verbal autopsy data, household surveys, disease registries, health service use, disease notifications, and other sources. According to the WHO, the underlying cause of death is defined as ‘the disease or injury which initiated the train of morbid events leading directly to death, or the circumstances of the accident or violence which produced the fatal injury’ [11]. Cholera death rates were calculated using the Cause of Death Ensemble model. Cholera deaths were adjusted to the GBD standard population estimates. Estimates of the GBD 2019 comply with the Guidelines for Accurate and Transparent Health Estimates Reporting (GATHER) statement [12].

Mortality of cholera is presented globally and within six WHO regions: Africa, the Americas, South-East Asia, Europe, Eastern Mediterranean, and Western Pacific. For this objective, the age-standardized mortality rates (ASRs, expressed per 100,000 persons) were used. Moreover, specific (by age and sex) mortality rates (expressed per 100,000 persons) were presented: the subgroup analyses were performed, with the age groups divided into four strata (<5/5–19/20–54/55+ years), for males and females separately. Additionally, trends in mortality rates of cholera in both sexes were presented on the global level and by WHO regions during the observed period.

### 2.3. Statistical Analysis

The joinpoint regression analysis (software version 4.9.0.0 from the Surveillance Research Program of the National Cancer Institute, Statistical Research, and Applications Branch, National Cancer Institute, Bethesda, MD, USA) [13] was utilized to evaluate mortality trends of cholera from 1990 to 2019. The joinpoint regression analysis makes it possible to identify the occurrence of possible joinpoint(s), i.e., year(s) where a statistically significant change in trend occurred [13]. A grid search method was used to determine whether rate changes are best described by a straight line (0 joinpoints) or by 1 or more linear segments (≥1 joinpoints) [14]. For the values of the dependent variable, the maximum number of joinpoints was 5 [13]. The trend was expressed as the Annual Percent Change (APC) of each trend segment and the Average Annual Percent Change (AAPC) over the entire considered period, with their corresponding 95% Confidence Intervals (CIs) [15]. The Monte Carlo permutation method (through 4499 permutations for different samples of each year’s rate) was used to assess whether APC/AAPC was significant (*p* < 0.05), and the trend was described as ‘‘significantly increased’’ or ‘‘significantly decreased’’ when the APC/AAPC was positive or negative, respectively [13]. For statistically non-significant trends (when the APC/AAPC value was not significant, *p* ≥ 0.05), the term ‘‘stable’’ was used. Additionally, a test for parallelism and a test of coincidence were used for the model to evaluate whether trends are parallel or coincident [16].

### 2.4. Ethical Considerations

The study was approved by the Ethics Committee of the Faculty of Medical Sciences, University of Kragujevac (Ref. No.: 01-14321, 13 November 2017), entitled “Epidemiology of the most common health disorders. “The data are fully aggregated, without any identification data, and no patient approvals were required for the study.

## 3. Results

**Figure 1:**
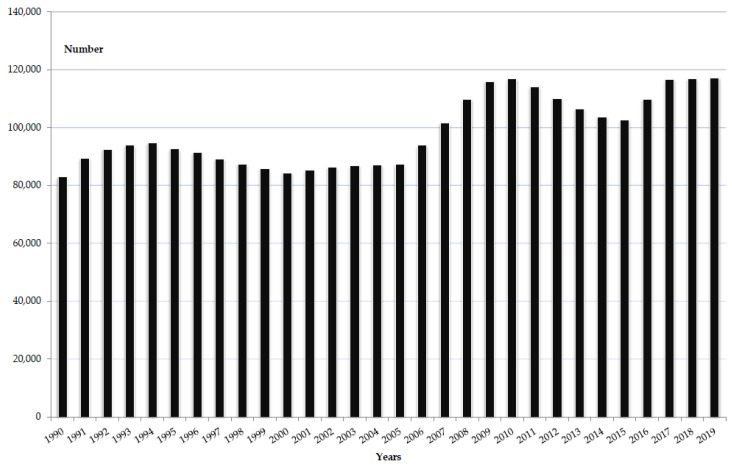
The number of cholera deaths in the world, 1990–2019 (Source of data Ref. [10]).

Over the 1990–2019 period, the number of deaths due to cholera in both sexes together was increasing worldwide (Figure 1). Per annum, the number of cholera-related deaths ranged from 83,045 in 1990 to 117,167 in 2019. During the observed period, there were about 3.0 million deaths due to cholera in the world.

**Figure 2:**
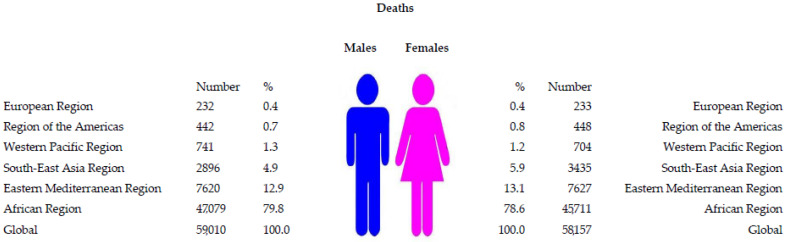
The number of cholera deaths, by regions of World Health Organization and sexes, 2019 (Source of data Ref. [10]).

In both sexes in 2019, most cholera-related deaths (92,790; 79.2% of the total) were recorded in the African Region, followed by the Eastern Mediterranean Region (15,247; 13.0%) (Figure 2). The differences in cholera deaths by region between males and females are less obvious. Less than 1% of the total cholera deaths occurred in the Region of Americas and European Regions.

**Figure 3:**
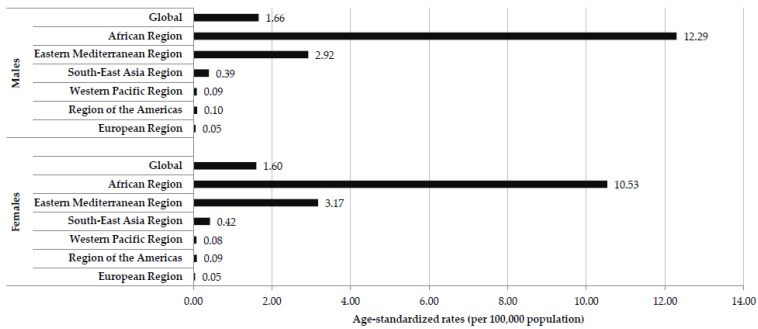
Mortality of cholera (global and by regions of World Health Organization), by sex, 2019 (Source of data Ref. [10]).

In 2019, the global age-standardized mortality rate from cholera was 1.66/100,000 population in males and 1.60/100,000 population in females (Figure 3). The differences in age-standardized rates of cholera mortality by regions between males and females are not striking. The highest rates were found in the African Region (12.29/100,000 in males and 10.53/100,000 population in females), followed by Eastern Mediterranean Region (2.92/100,000 population in males and 3.17/100,000 population in females). Mortality rates less than 0.5/100,000 population were reported both in males and females in Southeast Asia, Western Pacific, the Americas, and European regions.

**Figure 4:**
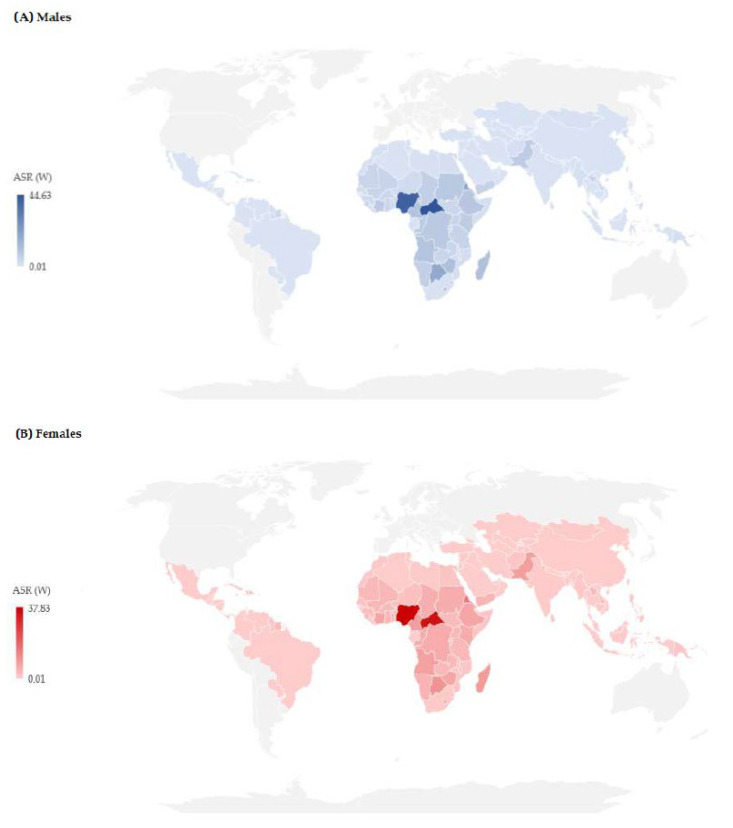
Estimated age-standardized rates (ASRs, per 100,000 population) of cholera mortality by countries and by sex ((**A**) Males; (**B**) Females), 2019 (Source of data Ref. [10]).

In 2019, the differences in age-standardized rates of mortality due to cholera in the world were conspicuous (Figure 4). In males, the cholera mortality rate was the highest in Central African Republic (44.63/100,000) and Nigeria (40.74/100,000), followed by Eritrea (20.23/100,000) and Botswana (18.39/100,000) (Figure 4A). In 61 countries, not a case of death from cholera was registered in males in 2019. Moreover, there was a significant variation in age-standardized mortality rates due to cholera in females across the countries: the highest mortality rate was in Nigeria (37.83/100,000) and Central African Republic (34.64/100,000), followed by Eritrea (15.82/100,000) (Figure 4B). In 60 countries, not a case of death from cholera was registered in females in 2019.

**Figure 5:**
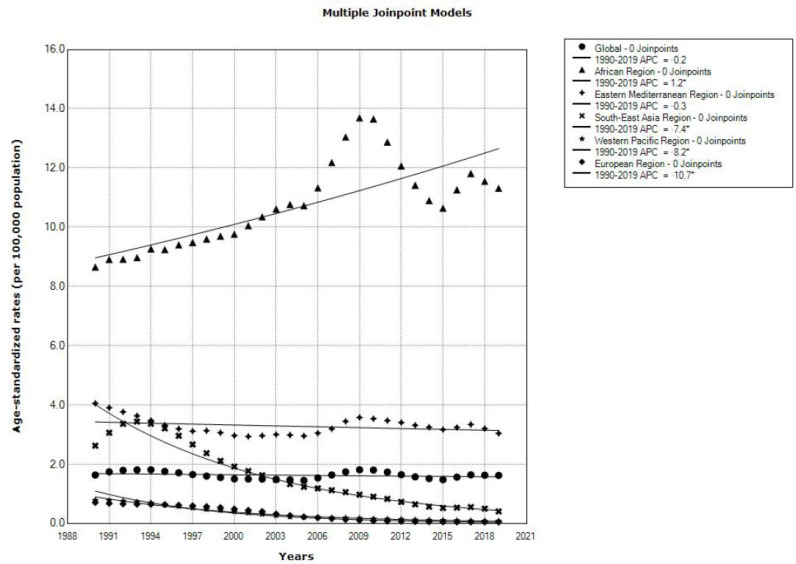
Joinpoint regression analysis of cholera mortality (global and by regions of World Health Organization), 1990–2019. * Statistically significant trend (*p* < 0.05). (Source of data Ref. [10]).

Globally, the trend for cholera-related mortality rates in both sexes together from 1990 to 2019 was stable (by −0.2% per year, 95% CI = −0.5 to 0.1) (Figure 5). When the cholera mortality trend was analyzed by six WHO regions, significantly decreasing trends were observed in three regions, the European Region (by −10.7% per year, 95% CI = −11.6 to −9.8), the Western Pacific Region (by −8.2% per year, 95% CI = −8.5 to −8.0), and the South-East Asia Region (by −7.4% per year, 95% CI = −7.8 to −6.9). In the African Region, a significantly increasing cholera-related mortality trend was observed (by 1.2% per year, 95% CI = 0.8 to 1.6); mortality rates increased from 8.7/100,000 in 1990 to 11.3/100,000 in the last year observed and peaked at 13.7 per 100,000 in 2009 and 2010. In the Eastern Mediterranean Region, a stable trend for cholera mortality rates was determined (by −0.3% per year, 95% CI = −0.7 to 0.1). Joinpoint results are not shown for mortality in the Region of the Americas because no case of cholera death occurred in at least one year in the observed period.

**Figure 6:**
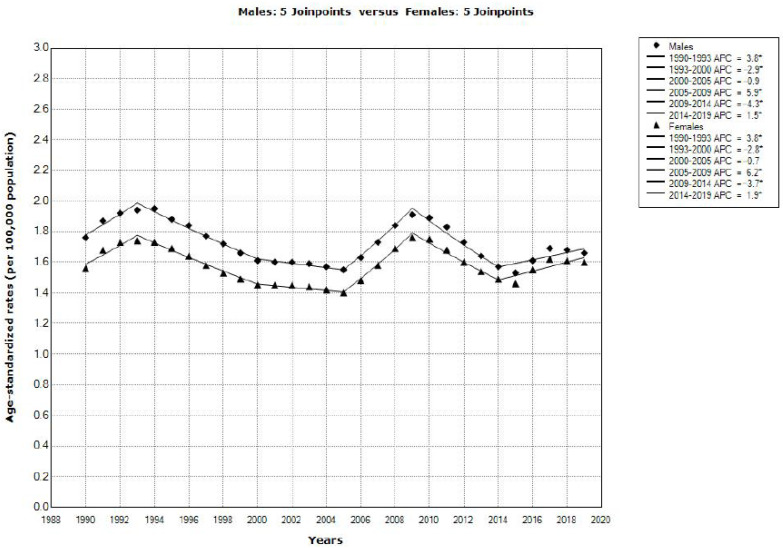
Joinpoint regression analysis of global cholera mortality, by sexes, 1990–2019. * Statistically significant trend (*p* < 0.05). (Source of data Ref. [10]).

**Figure 7:**
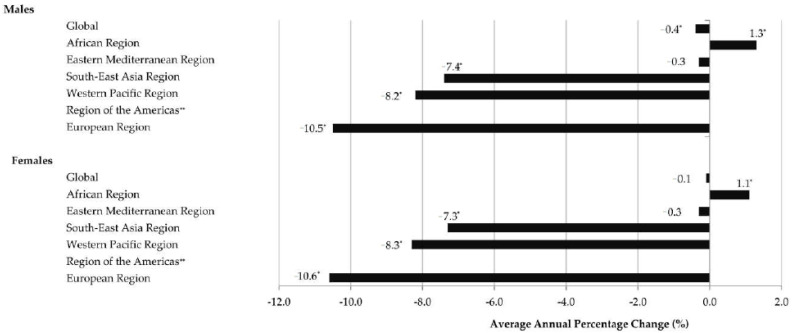
Trends in cholera mortality (age-standardized rates, per 100,000 population), by regions of World Health Organization and by sex, 1990–2019: a joinpoint regression analysis. * Statistically significant trend (*p* < 0.05); ** Joinpoint results are not shown for mortality in the region because no case of cholera death occurred in at least one year in the observed period. (Source of data Ref. [10]).

Globally, cholera-related mortality significantly decreased in males (by −0.4% per year, 95% CI = −0.7 to −0.1), while a stable trend was observed in females (by −0.1% per year, 95% CI = −0.4 to 0.2) in the observed period (Figure 6 and Figure 7). Joinpoint analyses of mortality both in males and females identified five joinpoints in the years 1993, 2000, 2005, 2009, and 2014, with six trends. The first, fourth, and sixth periods in males showed significantly increasing trends, with APC of 3.8%, 5.9%, and 1.5%, respectively (Figure 6). In males, the trends from 1993 to 2000 and from 2009 to 2014 showed a significant decrease (with APC of −2.9% and −4.3%, respectively). The first, fourth, and sixth periods in females showed significantly increasing trends, with APC of 3.8%, 6.2%, and 1.9%, respectively. In females, the trends from 1993 to 2000 and from 2009 to 2014 showed a significant decrease (with APC of −2.8% and −3.7%, respectively). In both males and females, cholera-related mortality trends from 2000 to 2005 were stable, with APC of −0.9% and −0.7%, respectively. According to the comparability test, the trends in cholera-related mortality in males and females were not parallel and not coincident (*p* < 0.05). 

In both males and females, trends for cholera-related mortality rates from 1990 to 2019 showed significantly decreasing trends in three regions: in the European Region (AAPC = −10.5% and AAPC = −10.6%, respectively), the Western Pacific Region (AAPC = −8.2% and AAPC = −8.3%, respectively) and the South-East Asia Region (AAPC = −7.4% and AAPC = −7.3%, respectively) (Figure 7). In the African Region, significantly increasing cholera-related mortality trends were observed both in males and females (AAPC = 1.3% and AAPC = 1.1%, respectively). In the Eastern Mediterranean Region, stable trends for cholera mortality rates were observed both in males and females (equally, AAPC = −0.3%). Joinpoint results are not shown for mortality in the Region of the Americas because no case of cholera death occurred in at least one year in the observed period.

Cholera-related mortality rates decreased with age, both in males and females (Table 1). In both sexes, cholera mortality rates were almost five times higher in people aged under 20 years than in people aged 20 years or older. Both in males and females, a significant increase in mortality due to cholera was observed in younger age groups (<5 years and 5–19 years). Among 20–54 years old males and females, trends of the cholera mortality rates were stable. There were significantly decreasing trends in mortality for cholera among males and females aged 50+ years (equally, AAPC = −1.4%).

Globally, although a stable trend in mortality of cholera was observed worldwide, a significantly increased mortality trend was determined in the younger group (0–19 years) in the observed period, while a decreasing trend in mortality was recorded at the 20 and above years (Table 2). In both age groups (0–19 and ≥20 years), the African Region showed significantly increasing trends in the mortality of cholera. Both age groups (0–19 years and 20 and above years) generally showed favorable trends in the mortality due to cholera in the European Region, Western Pacific Region, and South-East Asia Region. Similarly, a significantly favorable trend in mortality of cholera at the young age group (0–19 years) was observed in the Eastern Mediterranean Region; in contrast, their older compatriots showed a stable trend.

## 4. Discussion

This study presented global trends in mortality due to cholera in the last three decades. During the observed period, there were about 3.0 million deaths due to cholera in the world. In 2019, most cholera-related deaths (nearly 80% of the total) were recorded in the African Region. The global trend for cholera mortality rates in both sexes together from 1990 to 2019 was stable. It is promising that the global trends in cholera mortality rates have been significantly decreasing in the last three decades in men. In both sexes, cholera mortality rates were the highest in the population under 5 years, with a significantly increasing trend observed. Despite the decrease in trends in mortality of cholera in most of the regions, observed in both sexes and in all age groups, in the African Region, increasing trends of cholera mortality rates were reported in both sexes and in all age groups.

In 2019, an estimated total of 117,167 cholera-related deaths occurred in the world (59,010 males and 58,157 females), with an ASR of 1.66 per 100,000 in males and 1.60 per 100,000 in females. Globally, compared to 1990, in 2019, there were approximately 34,000 more deaths from cholera. Despite the efforts to control the disease, cholera continues to occur as a large public health issue in many countries with limited resources in the last decades. Two substantial upsurges in the number of deaths from cholera in the world were observed in several successive years, around 2010 and 2018. Namely, in four consecutive years (since 2007), the number of cholera-related deaths has been increasing worldwide and almost doubled in 2010 due to large epidemics in several countries (including Haiti, Nigeria, Cameroon, Chad, the Democratic Republic of Congo) [5,17,18,19,20]. In Nigeria, during the whole period observed, intermittent outbreaks have been occurring, but 2010 was marked with a severe outbreak characterized by the highest case-fatality rate: in total, it was 4.5%, while the Nigerian states with the highest case-fatality rates in the 2010 outbreak were Plateau, Kaduna, and Katsina with rates 23.0%, 9.0%, and 7.6%, respectively [21]. The case-fatality rates in Nigeria could be in part due to changes in Vibrio cholerae infectivity (a highly virulent multidrug resistant atypical O1 El Tor biotype and non-O1/non-O139 Vibrio cholerae strains) that were disseminated across the country by human travel [21,22]. Although epidemiologic information on cholera transmission was sometimes absent or very limited, there was a clear link between outbreaks in Nigeria poverty and a lack of good water sources [22]. The principal mode of transmission, however, remains through contaminated water or food, and it is speculated that the hand-dug wells and contaminated ponds being relied on by most of the Nigerian states as sources of drinking water were a major transmission route during the outbreak in 2010. At the same time, in the Democratic Republic of Congo, a country with a civil war and disorganized healthcare infrastructure, the spatial distribution of cases showed that lake areas were the source of frequent epidemics that sometimes spread to big cities hundreds of kilometers away via traders and other travelers [17,20]. In 2010, probably for the first time in history, cholera appeared in Haiti and has become the largest epidemic of cholera in the last decades (with 476,714 recorded cases and 6648 deaths during just 1 year after the epidemic started) [17,23]. The initial spread of cholera throughout Haiti correlated in time and place with the arrival of a peacekeeper battalion during a UN troop rotation coming from Nepal, which was experiencing a cholera outbreak at that time. At the same time, Haiti’s deadly explosive cholera epidemic provoked panic that caused a migration of people to nearby areas and, consequently, a spill-over of the cholera outbreak to the neighboring Dominican Republic.

During the following years, complex emergencies enabled the exacerbation of an ongoing countrywide cholera outbreak (driven by water supply interruptions, high population densities, and population movement, resulting that the Democratic Republic of the Congo accounting for 5–14% of total cholera cases worldwide annually, with case-fatality rates higher in non-endemic areas and in the early phases of the outbreaks, possibly reflecting low levels of immunity and less appropriate prevention and treatment [6,20]. During the 2014 outbreak in Ghana [24], the fatality rate remained below the WHO target of 1%, but mortality data must be interpreted with caution, as in resource-limited settings, many deaths may occur before patients can reach the hospital for treatment or may have avoided notification/reporting [25]. For example, during a cholera outbreak in Kenya during civil unrest in 2008, an active community-based case-finding showed that 200% more fatal cases were found than reported, raising the estimated case-fatality rate from 5.5% to 11.4% [26]. Home antibiotic treatment, hospitalization, treatment in government-operated health facilities, and receiving education about cholera by health workers were protective against death, while the high fatality rates among hospitalized cases were associated with inadequate intravenous and oral hydration and substantial staff and supply shortages at the time of admission [26].

In contrast to the global trend of decreasing mortality, the WHO African region has been experiencing a rise in cholera cases in the last three decades. In 2019, Africa accounted for about 90% of the world’s cholera deaths. Namely, conditions favoring cholera transmission are common in Africa (such as inadequate access to safe water, poor sanitation facilities, poverty, and low vaccination rates), although there may also be certain unique environmental factors promoting cholera outbreaks in Africa (as indicated by the epidemiological link between exposure to lakes and cholera in some African countries), and all of that fueled by climate change and political instability [6,25,27,28,29]. Some authors suggest that the aquatic environmental reservoirs (such as algae, crustaceans, phytoplankton, and zooplankton) likely provide to the persistence of Vibrio cholerae in lakes and rivers, contributing to the outbreak and spread of cholera [27]. On the other hand, the highest case-fatality rates for cholera are reported in some countries in Africa (Nigeria, Cameroon, Chad, the Democratic Republic of Congo), which could be due to the lack of adequate resources, health care infrastructure, availability of treatment methods [1,20,21,22]. It is particularly worrying that the highest mortality rates with an unfavorable upward trend in cholera mortality are recorded among children under five in the African region. Since contaminated water is the main path for the transmission of cholera, children are particularly vulnerable to the risk of disease/outcomes: in younger people, insufficiently adopted hygiene habits are common, which with inadequate living conditions and diet, malnutrition, along with other diseases accompanied by a decrease in immunity or hypochlorhydria (e.g., with Helicobacter pylori infection, which is endemic in countries in Africa) affects the mortality of cholera in children under 5 [30,31,32]. Besides that, oral vaccines against cholera administered to children in lower- and middle-income countries do not induce the same immune responses as they do in developed countries, which can partly be explained by maternal antibody interference, malnutrition, and previous exposure to other agents [33]. Additionally, the true magnitude of the cholera burden in the African region remains insufficiently known (due to inadequate laboratory and epidemiological surveillance systems), whereby WHO estimates that the officially reported cases represent only 5–10% of the actual number occurring annually worldwide [1,4,9,25].

Widespread destruction during the Yemeni Civil War (2014–2019) triggered the world’s largest documented cholera epidemic of modern times [34]. In 2017 and 2019, war-torn Yemen accounted for 84% and 93% of all cholera cases in the world, with children constituting the majority of reported cases [1,34]. The case fatality risk was 0.22%, particularly in people younger than 5 years, as well as in people with poor nutrition status, with less access to food and purchasing power [34,35,36,37]. Namely, in approximately half of all children under five, a high prevalence of chronic malnutrition was reported, which lowered their resilience toward cholera [35,36,37].

Very low child and low adult mortality due to cholera characterized almost all developed countries (with greater than 95% access to improved sanitation infrastructure and healthcare facilities), with the probability of cholera endemicity assumed to be zero and the ability for secondary transmission of imported cases also assumed to be zero [6]. In contrast, high child and very high adult mortality characterized developing countries facing cholera epidemics triggered by overcrowding, poverty, insufficient water and sanitation facilities, movement or displacement of people, climate changes, insufficient healthcare facilities, and lower availability of preventive and therapy resources.

A global strategy on cholera control ‘Ending Cholera: The Global Roadmap to 2030’ (which was launched in 2017 by the Global Task Force on Cholera Control, endorsed in 2018 by the World Health Assembly) marks a new initiative for a unified approach to cholera prevention and control, targeting reducing cholera deaths by 90% and eliminating local cholera transmission in as many as to 20 countries by 2030 [4,38,39]. While significant progress has been made in the developed countries towards reducing cholera mortality through improvements in case management (by rehydration therapy, treatment with antibiotics, and use of oral cholera vaccines) and by improving sanitation and hygiene conditions, areas of high mortality remain in the African and the Eastern Mediterranean Region.

Cholera remains a disease that affects people mainly in low-income countries, mostly natives, as well as people visiting those countries (such as travelers, humanitarian people, and healthcare workers). Conversely, a case of imported cholera in high-income countries rarely leads to the spread of cholera in those countries, confirming that inequality in socioeconomic factors plays an important role in the transmission of the disease [2,6,40,41]. Among the population of the non-vaccinated communities in Bangladesh, people living in a household without a concrete floor, in an area with high population density, or those not treating drinking water were at a significantly higher risk for both cholera and cholera with severe dehydration [40]. A recent meta-analysis showed that factors associated with symptomatic cholera included education less than secondary level, unimproved water source, open container water storage, consumption of food outside the home, household contact with cholera, lack of water treatment, lack of handwashing, lack of income/wealth, infant breastfeeding status [41]. Vaccination, as a well-established means for the prevention of cholera, joined with a thorough implementation of safe drinking water, sanitation, and hygiene strategies, are crucial to cholera control and human health and well-being [1,2,3,4,42].

Although cholera was a disease notifiable to WHO till 2005, many countries underreported or failed to report it at all for various social and economic reasons. The unfavorable trends in mortality emphasize the need for a more effective public health approach to the prevention of cholera worldwide, as well as the need for further intensive international support and cooperation in order to achieve the set goals linked with the reduction in cholera deaths until 2030, especially in countries with limited resources [43,44,45].

### Strengths and Limitations of the Study

This study presented up-to-date global trends in mortality due to cholera in the last three decades. The presented trends are important not only for monitoring and assessment of cholera mortality in the world but also for the evaluation of preventive measures. Moreover, this study represents a substantial improvement from some previous studies since it described global and regional trends over three decades, the distribution of cholera throughout the world in 2019, with the joinpoint regression analysis used in trends assessment.

However, several sources of limitations in this study should be taken into account. Firstly, the accuracy of all estimates generally depends on the availability and quality of data. Further, a limitation specific to evaluating cholera-related deaths can lead to underestimation or under-reporting of mortality of cholera depending on the development and availability of health services. Finally, in this study, a descriptive epidemiological study design was used and could not investigate individual factors that contributed to the changes in trends of cholera mortality. Despite these shortcomings, this study provides useful insights into the variations in the mortality of cholera worldwide and could provide help for health authorities and policymakers to develop more effective cholera prevention strategies based on reliable estimates of the mortality of cholera.

## 5. Conclusions

Despite the decline in cholera mortality in most areas of the world during the last decades, cholera presents one of the main health challenges worldwide. Globally, cholera mortality rates were the highest in the population aged under 5 years in both sexes, with significantly increasing trends registered in the observed period. Unfortunately, the mortality of cholera showed an increasing trend in both sexes and in all ages in the African Region. Further consistent implementation of comprehensive cholera prevention and control measures involving the efforts of all segments of society could lead to a reduction in cholera mortality, especially in poverty-stricken countries and the most vulnerable populations.

## Figures and Tables

**Table 1:** Joinpoint regression analysis: global trends in age-specific mortality rates of cholera (per 100,000 population), by sex, 1990–2019.

	Males			Females		
Age (Years)	Age-Specific Rates		AAPC (95% CI)	Age-Specific Rates		AAPC (95% CI)
	1990	2019		1990	2019	
<5	7.3	8.4	+0.5 * (0.1 to 0.8)	7.2	8.4	+0.6 * (0.3 to 0.9)
5–19	0.1	0.2	+1.4 * (0.9 to 1.9)	0.2	0.3	+1.5 * (1.0 to 2.1)
20–54	0.4	0.4	−0.1 (−0.5 to 0.3)	0.4	0.6	−0.2 (−0.5 to 0.2)
55+	1.9	1.4	−1.4 * (−1.6 to −1.1)	1.5	1.1	−1.4 * (−1.7 to −1.1)

* Statistically significant trend; for the full period presented AAPC (Average Annual Percentage Change); CI = Confidence Interval. (Source of data Ref. [10]).

**Table 2:** Joinpoint regression analysis: Global trends in mortality rates of cholera in both sexes (per 100,000 population), by age (age strata: 0–19 and 20 and above years), and by regions of World Health Organization, 1990–2019.

Locations	Age					
	0–19 Years			20 and above Years		
	Age-Specific Rates		AAPC (95% CI)	Age-Specific Rates		AAPC (95% CI)
	1990	2019		1990	2019	
Global Regions	2.3	2.6	+0.5 * (0.1 to 0.9)	0.5	0.4	−0.7 * (−1.0 to −0.4)
African Region	6.5	9.8	+1.5 * (1.1 to 1.9)	2.9	3.3	+0.7 * (0.3 to 1.0)
Eastern Mediterranean Region	1.7	0.2	−1.5 * (−1.9 to −1.0)	0.9	0.8	+0.4 (−0.0 to 0.8)
South-East Asia Region	1.4	0.1	−7.9 * (−8.3 to −7.5)	0.7	0.2	−6.1 * (−6.6 to −5.7)
Western Pacific Region	1.4	0.9	−8.6 * (−9.1 to −8.0)	0.1	0.1	−6.5 * (−7.0 to −6.1)
Region of the Americas	0.0	0.1	-- **	0.0	0.1	-- **
European Region	1.5	0.1	−11.6 * (−12.4 to −10.8)	0.1	0.1	−4.9 * (−5.5 to −4.3)

* Statistically significant trend (*p* < 0.05); ** Joinpoint results are not shown for mortality in the region because no case of cholera death occurred in at least one year in the observed period; for the full period presented AAPC (Average Annual Percentage Change); CI = Confidence Interval. (Source of data Ref. [10]).

## Data Availability

Data is contained within the article.
